# Pattern of psychiatric in-patient admissions in Al Ain, United Arab Emirates

**DOI:** 10.1192/bji.2020.54

**Published:** 2021-05

**Authors:** Karim Abdel Aziz, Dina Aly El-Gabry, Mouza Al-Sabousi, Ghanem Al-Hassani, Moataz M. Ragheb, Mohamed Elhassan Elamin, Mohamed Abdel-Maksoud, Emmanuel Stip, Aidroos Al-Aidroos, Tareq Al-Shehhi, Danilo Arnone

**Affiliations:** 1 Assistant Professor of Psychiatry and Consultant Psychiatrist, Department of Psychiatry, College of Medicine and Health Sciences, United Arab Emirates University, United Arab Emirates. Email: danilo.arnone@uaeu.ac.ae; 2 Associate Professor of Psychiatry and Consultant Psychiatrist, Okasha Institute of Psychiatry, Neuropsychiatry Department, Faculty of Medicine, Ain Shams University, Egypt; 3 Resident in Psychiatry, Behavioural Science Institute, Al Ain Hospital, United Arab Emirates; 4 Associate Professor of Psychiatry and Consultant Psychiatrist, Department of Psychiatry, Paul L. Foster School of Medicine, Texas Tech University Health Sciences Center, El Paso, Texas, USA; 5 Registrar in Psychiatry, Department of Psychiatry, Highfield Healthcare, Dublin, Ireland; 6 Maudsley Health Dubai, Al Amal Psychiatric Hospital, Al Awir, Dubai, United Arab Emirates; 7 Professor of Psychiatry and Consultant Psychiatrist, Centre Hospitalier Universitaire de Montreal (CHUM), Institute Universitaireen Santé Mentale de Montréal, Université de Montreal, Canada; 8 Associate Professor of Psychiatry and Consultant Psychiatrist; 9 General Practitioner, Institute of Psychiatry, Psychology and Neuroscience, Department of Psychological Medicine, Centre for Affective Disorders, King's College London, UK

**Keywords:** United Arab Emirates, psychiatry, psychosis, bipolar disorder, substance misuse

## Abstract

An understanding of the current state of mental health services in the United Arab Emirates (UAE) from a clinical perspective is an important step in advising government and stakeholders on addressing the mental health needs of the fast-growing population. We conducted a retrospective study of data on all patients admitted to a regional psychiatric in-patient unit between June 2012 and May 2015. More Emiratis (UAE nationals) were admitted compared with expatriates. Emiratis were diagnosed more frequently with substance use disorders and expatriates with stress-related conditions. Psychotic and bipolar disorders were the most common causes for admission and had the longest in-patient stays; advancing age was associated with longer duration of in-patient stay.

Mental illness is a leading cause of suffering and disability in the world. Global estimates predict an increasing impact in the next decades.^1^ There is an urgent need for the United Arab Emirates (UAE) to ascertain the current level of mental health services to plan effectively for a rapidly expanding population. Currently, the UAE absorbs 5.54% of the world mental health burden, but the country publishes only 1% of peer-reviewed mental health research.^2^

The UAE is a multicultural federation of seven emirates, consisting of Abu Dhabi, Ajman, Dubai, Fujairah, Ras Al Khaimah, Sharjah and Umm Al Quwain (Fig. 1). UAE psychiatric facilities include 25 psychiatric beds in general hospitals and 80 in specialised units, equivalent to 0.9 hospital beds/100 000 people, lower than other Arab countries (e.g. Morocco, 4.1/100 000) and Western countries (e.g. UK, 23.9/100 000; USA, 18.6/100 000; Australia, 7.2/100 000) (www.who.int/en/).

**Fig. 1:**
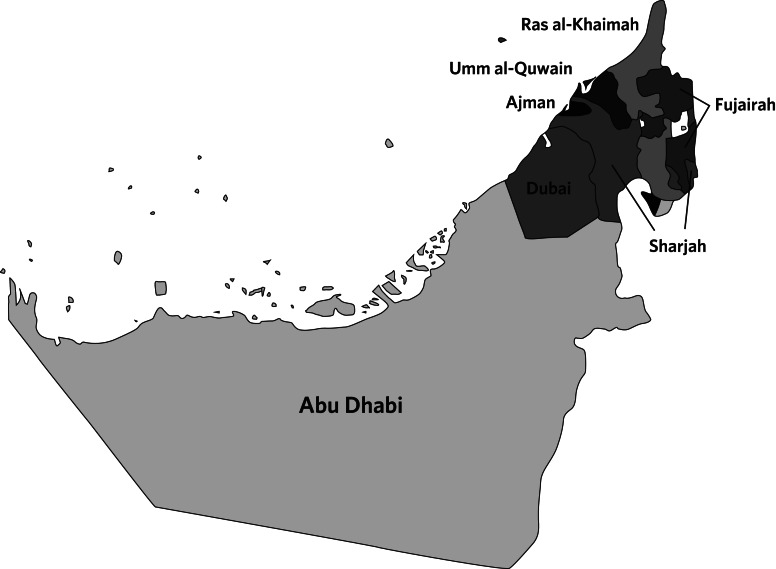
Map of the seven emirates in the United Arab Emirates (courtesy of CK-12: https://www.ck12.org/user:zxbpc2r3z0blcglzzc5vcmc./book/episd-world-geography-2017-2018/section/6.4/).

In mid 2016 the population of the emirate of Abu Dhabi was 2.9 million, out of a total population of the UAE of 9.3 million as estimated in 2017 (https://u.ae/en/about-the-uae/fact-sheet). In this paper, we retrospectively reviewed the pattern of admission in the 33-bed psychiatric unit based in Al Ain hospital, the largest psychiatric in-patient unit in the eastern region of the emirate of Abu Dhabi, which absorbs 30% of the national capacity.^3^

## Method

We conducted a retrospective study by collecting data on all patients admitted to the psychiatric in-patient unit between June 2012 and May 2015. This in-patient provision is mixed for Emiratis (UAE nationals) and expatriates. Data were obtained from the computerised patient medical record system and included patients of all ages, genders, nationalities, clinical diagnoses according to ICD-10^4^ and length of admission. Al Ain hospital's ethics committee approved the study.

Parametric and non-parametric data were analysed using SPSS version 24.0 for Windows. Chi-squared or Fisher's exact tests, independent-sample Student's *t*-tests or Mann–Whitney *U*-tests were used as appropriate. Pearson Correlation Test (*r*) and Spearman’s rho Correlation Test (*r*_s_) for studying the direction and power of relationships of variables was used for correlational analyses of quantitative data. The statistical threshold for significance was set at α = 0.05.

## Results

Over the 3-year period of the study, 961 patients were admitted; 546 were male (56.8%), 415 were female (43.2%); the mean age was 32.49 years (s.d. = 11.72). The mean number of days in hospital was 14.52 (s.d. = 27.45). Table 1 shows the frequencies of diagnoses and length of admission. The most frequent diagnoses were psychotic disorders, bipolar disorder and substance use disorders, and these were also associated with the longest duration of admission. There was a significant positive correlation between duration of stay and increasing age (*r* = 0.09, *P* = 0.005) but no significant relationship with gender (*r* = 0.025, *P* = 0.44).

**Table 1:** Pattern and frequencies of diagnoses in the total sample (*n* = 961)

Diagnosis	*n* (%)	Length of stay, days
Psychotic disorders	301 (31.3)	22
Bipolar disorder	200 (20.8)	18.55
Substance use disorders	121 (12.6)	5.1
Major depressive disorder	90 (9.4)	12.33
Acute stress disorder	59 (6.1)	7.6
Adjustment disorder	54 (5.6)	9.7
Other disorders	44 (4.6)	8.9
Anxiety disorders	31 (3.2)	5.3
Aggressive/suicidal behaviour	23 (2.4)	6.3
Personality disorders	22 (2.3)	5.6
Organic mental disorders	16 (1.7)	6.4

Of the total, 570 patients (59.3%) were foreign expatriates (from 42 different countries) and 391 (40.7%) were local Emiratis. The most common non-Emirati groups admitted were Ethiopians (9.9%), followed by Pakistanis (7.4%), Omanis (5.6%), Indians (5.4%) and Bengalis (5.3%). Frequency of all nationalities and reasons for admission are presented in supplementary Table 1, available at https://doi.org/10.1192/bji.2020.54. In both Emiratis and expatriates, psychotic disorders were the most common condition. Ethiopians presented with an acute stress reaction in 29% of cases, followed by 26% with psychotic disorders and 17% with bipolar disorder. In the second most frequent group, from Pakistan, psychotic disorders (22%) and bipolar disorder (21%) were the most common diagnoses. Omanis presented most frequently with a psychotic disorder (44%), as did Palestinians (64%). Other interesting trends included major depressive disorder in 47% of Afghani patients and psychotic disorders in 45% of Bangladeshi patients. Indians presented most frequently with psychotic disorders and bipolar disorders (31% each).

A comparison between local and non-local patients indicated that Emiratis were on average 3 years older. Substances use disorders were more frequent in Emiratis and acute stress reactions and adjustment disorders in the expatriates (Tables 2 and 3).

**Table 2:** Comparison of Emiratis and expatriates

	Emiratis (*n* = 391)	Expatriates (*n* = 570)	Test	*P*
Gender, male:female	216:175	330:240	χ^2^ = 0.665	0.415
Age, years: mean (s.d.)	34.34 (12.665)	31.23 (10.853)	*t* = 4.073	<0.0001*
Duration of stay, days: mean (s.d.)	15.19 (39.56)	14.07 (14.07)	*t* = 0.619	0.536

*t*, Student's *t*-test.

*Statistically significant.

**Table 3:** Pattern of diagnosis in Emiratis (*n* = 391) compared with expatriates (*n* = 570)

Diagnosis	Emiratis, *n*	Expatriates, *n*	χ^2^	*P*
Psychotic disorders	122	179	0.004	0.947
Bipolar disorder	75	125	1.063	0.303
Substance use disorders	90	31	65.120	<0.0001*
Major depressive disorder	34	56	0.348	0.555
Anxiety disorders	14	17	0.266	0.606
Personality disorders	13	9	3.160	0.075
Adjustment disorder	10	44	11.651	0.001*
Aggressive/suicidal behaviour	8	15	0.340	0.560
Organic mental disorders	7	9	0.063	0.801
Acute stress disorder	2	57	36.234	<0.0001*
Other	16	28	0.357	0.550

* Statistically significant.

## Discussion

To the best of our knowledge, this is the first study investigating the pattern of psychiatric in-patient admissions in the UAE. Psychotic disorders were the most common conditions in the in-patient population, and they involved the longest stay in hospital. Bipolar disorder and substance use disorders followed in terms of frequency and admission length. Increasing age was associated with longer duration of in-patient stay. Expatriates were a heterogeneous group mostly originating from the Middle East, North Africa and Asia. The most common non-Emirati groups were Ethiopians (9.9%), Pakistanis (7.4%), Omanis (5.6%), Indians (5.4%) and Bengalis (5.3%). Expatriate patients were on average 3 years younger than Emiratis. Although more Emiratis had a diagnosis of substances use disorder, expatriates were diagnosed more frequently with conditions associated with stress, namely reactions to acute stress and adjustment disorders.

There were more Emiratis admitted compared with patients from other countries. This might reflect the fact that 19% of the total Emirati population live in the region of Abu Dhabi (41% in Al Ain). Expatriates receive variable levels of health insurance coverage, which may not necessarily include mental healthcare. Expatriates are also screened on entry into the UAE for health problems. The older age of Emiratis at the time of hospital admission may be related to what is reported in the literature as service underutilisation^5^ fostered by a tendency to consult traditional healers as a preferred first option to treat mental illness,^6–8^ which might delay diagnosis and treatment.^9^ Delayed diagnosis in conditions such as schizophrenia and bipolar disorder are associated with increased severity, affecting hospital admissions and outcome.^10^ Stigma associated with mental illness is a recognised problem in the Arab world,^11^ which must be addressed to minimise suffering, premature death and large economic costs due to non-engagement.^1^

The United Nations World Drug Report 2019 suggests that the use of drugs is significant in the Middle East (wdr.unodc.org). Although the National Rehabilitation Centre in Abu Dhabi City is available for substance use disorders in the region,^12^ it is located far from Al Ain Hospital, explaining the number of cases admitted locally. Many expatriates originate from countries affected by political unrest, poverty, ethnic/racial discrimination and military conflicts. Added stressors, such as isolation and societal differences after moving to the UAE, can also occur more frequently, explaining the higher occurrence of stress-related diagnoses in expatriates.

The Global Health Observatory data repository of the World Health Organization indicates that there are only 1.6 psychiatrists, 0.76 psychologists, 0.36 social workers and 4.37 nurses per 100 000 people living in the UAE. This level of workforce is unlikely to provide a level of services comparable to Western countries such as the USA, where there are 10.5 psychiatrists per 100 000. The demand for mental health services in the Abu Dhabi region is likely to be over 100% higher by 2030, according to the Abu Dhabi Department of Health's Healthcare Capacity Masterplan (www.doh.gov.ae/). This forecast, based on the current level of resources, requires careful consideration for planning. A way forward may be to follow the World Psychiatric Association's guidance on community mental healthcare.^13,14^ This approach advocates that in case of limited resources, it is more effective to create a stepped-care approach, with diagnostic evaluation and treatment initiation in primary care under specialist supervision.^14^

### Limitations

Limitations of this study include the retrospective nature of the work, open to recall bias, and the limited availability of clinical data and records of level of insurance cover. We did not systematically record numbers/proportions of patients of Muslim faith and those who consult traditional healers, although 57.4% of the sample included nationalities of Islamic faith. Survey data from Al Ain suggest that only 38% of UAE citizens seek psychiatric help and prefer consulting traditional healers as a first step.^6,7^ The study does not cover the past few years of admissions, although we are not aware of any major societal or structural changes. We were unable to quantify the number of compulsory admissions. Currently in the UAE there is no equivalent to the UK's Mental Health Act. We only collected primary diagnoses. Information regarding secondary diagnoses might have been informative.^15^

### Implications

There is great need to improve knowledge about mental illness in the UAE and to develop ways of increasing access to mental health services. Our analysis provides clinical information that can contribute to promoting knowledge and help to reduce stigma by improving the perception of mental illness. It is likely that a screening programme based in primary care might facilitate earlier detection of mental illness in the community, while a proactive education programme in schools and in the community might prove useful to improve knowledge about mental disorders and how to access services at the earliest convenience. Future service planning would also benefit from creating specialised substance misuse services locally to serve the needs of the community.

## Data Availability

The study data are available on request.

## Supplementary material

For supplementary material accompanying this paper visit http://dx.doi.org/10.1192/bji.2020.54. click here to view supplementary material
