# Supplementary material for: Pattern of psychiatric in-patient admissions in Al Ain, United Arab Emirates
Source: BJPsych Int. 2021 May;18(2):46–50. doi: 10.1192/bji.2020.54 (PMC8274411; doi:10.1192/bji.2020.54)
Supplement: Supplementary file 1 [file S2056474020000549sup.zip › S2056474020000549sup002.docx]

| **N (%)** | **MDD** | **BD** | **PsychDis** | **SMD** | **AnxDis** | **PerDis** | **OMD** | **AD** | **AcStDis** | **AgSuBeh** | **Others** |  |
| --- | --- | --- | --- | --- | --- | --- | --- | --- | --- | --- | --- | --- |
| **Emirates** | 34 (8.6) | 75 (19) | 122 (31) | 90 (23) | 14 (3.5) | 13 (3.3) | 7 (1.8) | 10 (2.5) | 2 (0.5) | 8 (2) | 16 (4) | **391** |
| **Afghani** | 8 (47) | 1 (5.9) | 2 (12) | 0 (0) | 1 (5.9) | 0 (0) | 2 (12) | 1 (5.9) | 2 (12) | 0 (0) | 0 (0) | **17** |
| **American** | 1 (20) | 2 (40) | 0 (0) | 0 (0) | 0 (0) | 1 (20) | 0 (0) | 0 (0) | 1 (20) | 0 (0) | 0 (0) | **5** |
| **Bahraini** | 0 (0) | 2 (100) | 0 (0) | 0 (0) | 0 (0) | 0 (0) | 0 (0) | 0 (0) | 0 (0) | 0 (0) | 0 (0) | **2** |
| **Bangladeshi** | 5 (9.8) | 12 (23.5) | 23 (45) | 0 (0) | 2 (3.9) | 0 (0) | 0 (0) | 1 (2) | 6 (12) | 0 (0) | 2 (4) | **51** |
| **British** | 2 (67) | 0 (0) | 1 (33) | 0 (0) | 0 (0) | 0 (0) | 0 (0) | 0 (0) | 0 (0) | 0 (0) | 0 (0) | **3** |
| **Canadian** | 0 (0) | 0 (0) | 1 (100) | 0 (0) | 0 (0) | 0 (0) | 0 (0) | 0 (0) | 0 (0) | 0 (0) | 0 (0) | **1** |
| **Comorian** | 1 (17) | 0 (0) | 1 (17) | 2 (33) | 0 (0) | 0 (0) | 0 (0) | 0 (0) | 0 (0) | 0 (0) | 2 (33) | **6** |
| **Egyptian** | 3 (14) | 3 (14) | 7 (32) | 1 (4.5) | 0 (0) | 1 (4.5) | 1 (4.5) | 2 (9) | 1 (4.5) | 1 (4.5) | 2 (9) | **22** |
| **Ethiopian** | 4 (4.2) | 16 (16.8) | 25 (26.3) | 0 (0) | 3 (3.1) | 0 (0) | 0 (0) | 16 (16.8) | 28 (29.4) | 2 (2.1) | 1 (1) | **95** |
| **Filipino** | 6 (20) | 7 (23) | 9 (30) | 0 (0) | 0 (0) | 0 (0) | 0 (0) | 4 (13) | 3 (10) | 0 (0) | 1 (3.3) | **30** |
| **French** | 0 (0) | 1 (100) | 0 (0) | 0 (0) | 0 (0) | 0 (0) | 0 (0) | 0 (0) | 0 (0) | 0 (0) | 0 (0) | **1** |
| **German** | 0 (0) | 2 (100) | 0 (0) | 0 (0) | 0 (0) | 0 (0) | 0 (0) | 0 (0) | 0 (0 ) | 0 (0) | 0 (0) | **2** |
| **Ghanaian** | 0 (0) | 0 (0) | 0 (0) | 0 (0) | 0 (0) | 0 (0) | 0 (0) | 1 (100) | 0 (0) | 0 (0) | 0 (0) | **1** |
| **Indian** | 4 (7.6) | 16 (30.7) | 16 (30.7) | 4 (7.6) | 1 (1.9) | 0 (0) | 1 (1.9) | 4 (7.6) | 3 (5.7) | 1 (1.9) | 2 (3.8) | **52** |
| **Indonesian** | 0 (0) | 1 (9) | 3 (27) | 0 (0) | 0 (0) | 1 (9) | 0 (0) | 1 (9) | 2 (18) | 2 (18) | 1 (9) | **11** |
| **Iranian** | 1 (50) | 0 (0) | 0 (0) | 1 (50) | 0 (0) | 0 (0) | 0 (0) | 0 (0) | 0 (0) | 0 (0) | 0 (0) | **2** |
| **Iraqi** | 0 (0) | 2 (33) | 1 (17) | 0 (0) | 1 (17) | 0 (0) | 0 (0) | 1 (17) | 0 (0) | 0 (0) | 1 (17) | **6** |
| **Jordanian** | 1 (7.1) | 4 (28) | 7 (50) | 0 (0) | 0 (0) | 0 (0) | 1 (7.1) | 0 (0) | 0 (0) | 1 (7.1) | 0 (0) | **14** |
| **Lebanese** | 0 (0) | 0 (0) | 1 (33) | 0 (0) | 1 (33) | 1 (33) | 0 (0) | 0 (0) | 0 (0) | 0 (0) | 0 (0) | **3** |
| **Malawian** | 0 (0) | 0 (0) | 0 (0) | 0 (0) | 0 (0) | 0 (0) | 0 (0) | 1 (100) | 0 (0) | 0 (0) | 0 (0) | **1** |
| **Malaysian** | 0 (0) | 0 (0) | 0 (0) | 0 (0) | 0 (0) | 0 (0) | 0 (0) | 1 (100) | 0 (0) | 0 (0) | 0 (0) | **1** |
| **Mauritius** | 0 (0) | 0 (0) | 1 (100) | 0 (0) | 0 (0) | 0 (0) | 0 (0) | 0 (0) | 0 (0) | 0 (0) | 0 (0) | **1** |
| **Moroccan** | 3 (27) | 3 (27) | 3 (27) | 1 (9) | 0 (0) | 0 (0) | 0 (0) | 0 (0) | 1 (9) | 0 (0) | 0 (0) | **11** |
| **Nepalese** | 0 (0) | 2 (28) | 1 (14) | 1 (14) | 1 (14) | 0 (0) | 0 (0) | 1 (14) | 1 (14) | 0 (0) | 0 (0) | **7** |
| **New Zealander** | 1 (100) | 0 (0) | 0 (0) | 0 (0) | 0 (0) | 0 (0) | 0 (0) | 0 (0) | 0 (0) | 0 (0) | 0 (0) | **1** |
| **Omani** | 3 (5) | 6 (11) | 24 (44) | 12 (22) | 1 (2) | 1 (2) | 2 (4) | 1 (2) | 0 (0) | 2 (4) | 2 (4) | **54** |
| **Pakistani** | 7 (10) | 15 (21) | 16 (22) | 4 (6) | 3 (4) | 2 (3) | 2 (3) | 6 (8) | 5 (7) | 3 (4) | 8 (11) | **71** |
| **Palestinian** | 0 (0) | 1 (9) | 7 (63) | 1 (9) | 1 (9) | 0 (0) | 0 (0) | 0 (0) | 0 (0) | 0 (0) | 1 (9) | **11** |
| **Qatari** | 0 (0) | 1 (100) | 0 (0) | 0 (0) | 0 (0) | 0 (0) | 0 (0) | 0 (0) | 0 (0) | 0 (0) | 0 (0) | **1** |
| **Saudi** | 1 (12) | 2 (25) | 2 (12) | 2 (25) | 0 (0) | 0 (0) | 0 (0) | 0 (0) | 0 (0) | 1 (12) | 0 (0) | **8** |
| **Somali** | 0 (0) | 3 (27) | 5 (45) | 0 (0) | 0 (0) | 0 (0) | 0 (0) | 1 (9) | 0 (0) | 0 (0) | 2 (18) | **11** |
| **South African** | 0 (0) | 0 (0) | 0 (0) | 0 (0) | 0 (0) | 0 (0) | 0 (0) | 0 (0) | 0 (0) | 0 (0) | 1 (100) | **1** |
| **Sri Lankan** | 0 (0) | 0 (0) | 1(100) | 0 (0) | 0 (0) | 0 (0) | 0 (0) | 0 (0) | 0 (0) | 0 (0) | 0 (0) | **1** |
| **Sudanese** | 1 (4) | 12 (48) | 9 (36) | 0 (0) | 0 (0) | 1 (4) | 0 (0) | 0 (0) | 1 (4) | 0 (0) | 1 (4) | **25** |
| **Syrian** | 1 (7) | 6 (40) | 2 (13) | 0 (0) | 1 (7) | 1 (7) | 0 (0) | 0 (0) | 1 (7) | 2 (13) | 1 (7) | **15** |
| **Taiwan** | 1 (50) | 0 (0) | 1 (50) | 0 (0) | 0 (0) | 0 (0) | 0 (0) | 0 (0) | 0 (0) | 0 (0) | 0 (0) | **2** |
| **Tanzania** | 1 (50) | 0 (0) | 1 (50) | 0 (0) | 0 (0) | 0 (0) | 0 (0) | 0 (0) | 0 (0) | 0 (0) | 0 (0) | **2** |
| **Tunisia** | 0 (0) | 0 (0) | 0 (0) | 0 (0 ) | 0 (0) | 0 (0) | 0 (0) | 0 (0) | 1 (100) | 0 (0) | 0 (0) | **1** |
| **Uganada** | 0 (0) | 0 (0) | 1 (50) | 0 (0) | 0 (0) | 0 (0) | 0 (0) | 1 (50) | 0 (0) | 0 (0) | 0 (0) | **2** |
| **Yemeni** | 1 (6) | 5 (29) | 7 (41) | 1 (5.8) | 1 (6) | 0 (0) | 0 (0) | 1 (6) | 1 (6) | 0 (0) | 0 (0) | **17** |
| **Unknown** | 0 (0) | 0 (0) | 1 (50) | 1 (50) | 0 (0) | 0 (0) | 0 (0) | 0 (0) | 0 (0) | 0 (0) | 0 (0) | **2** |
|  | **90 (9)** | **200 (20)** | **301 (31)** | **121 (12)** | **31 (3)** | **22 (2)** | **16 (2)** | **54 (6)** | **59 (6)** | **23 (2)** | **44 (5)** | **961** |

**Supplementary Table1:** Detailed description of country of origin and diagnoses across the sample. MDD: Major Depressive Disorder; BD: Bipolar Disorder; PsychDis: Psychotic Disorders; SMD: Substance Misuse Disorders; AnxDis: Anxiety Disorders; PerDis: PD: Personality Disorders; OMD: Organic Mental Disorders; AD: Adjustment Disorder; AcStDis: Acute Stress Disorder; AgSuBeh: Aggressive/Suicidal Behavior.

*Percentages are rounded to whole numbers.*
